# SnO_2_-Based Ultra-Flexible Humidity/Respiratory Sensor for Analysis of Human Breath

**DOI:** 10.3390/bios13010081

**Published:** 2023-01-03

**Authors:** Moumita Deb, Mei-Yu Chen, Po-Yi Chang, Pin-Hsuan Li, Ming-Jen Chan, Ya-Chung Tian, Ping-Hung Yeh, Olivier Soppera, Hsiao-Wen Zan

**Affiliations:** 1 Department of Photonics, College of Electrical and Computer Engineering, National Yang Ming Chiao Tung University, 1001 Ta Hsueh Rd., Hsinchu 30010, Taiwan; 2 Department of Photonics, College of Electrical and Computer Engineering, National Chiao Tung University, 1001 Ta Hsueh Rd., Hsinchu 30010, Taiwan; 3 Department of Physics, Tamkang University, 151, Yingzhuan Rd., Tamsui, New Taipei City 25137, Taiwan; 4 Université de Haute-Alsace, CNRS, IS2M UMR 7361, F-68100 Mulhouse, France; 5 Université de Strasbourg, F-67081 Strasbourg, France; 6 Department of Medicine, Chang Gung University, Taoyuan 333, Taiwan; 7 Kidney Research Center and Department of Nephrology, Linkou Chang Gung Memorial Hospital, Taoyuan 333, Taiwan; 8 Graduate Institute of Clinical Medical Science, College of Medicine, Chang Gung University, Taoyuan 333, Taiwan

**Keywords:** SnO_2_, plastic wrap, NIR annealing, humidity sensor, breath analysis

## Abstract

Developing ultraflexible sensors using metal oxides is challenging due to the high-temperature annealing step in the fabrication process. Here, we demonstrate the ultraflexible relative humidity (RH) sensor on food plastic wrap by using 808 nm near-infrared (NIR) laser annealing for 1 min at a low temperature (26.2–40.8 °C). The wettability of plastic wraps coated with sol-gel solution is modulated to obtain uniform films. The surface morphology, local temperature, and electrical properties of the SnO_2_ resistor under NIR laser irradiation with a power of 16, 33, and 84 W/cm^2^ are investigated. The optimal device can detect wide-range RH from 15% to 70% with small incremental changes (0.1–2.2%). X-ray photoelectron spectroscopy reveals the relation between the surface binding condition and sensing response. Finally, the proposed sensor is attached onto the face mask to analyze the real-time human breath pattern in slow, normal, and fast modes, showing potential in wearable electronics or respiration monitoring.

## 1. Introduction

Relative humidity (RH) is the measurement of water vapor in air at a specific temperature and pressure [1]. Sensing relative humidity effectively is crucial in various fields such as agriculture [2], food packaging [3], industrial process control [4], aerospace [5], indoor climate control [6], health monitoring [7], etc. Humidity sensors also play a vital role in human respiratory monitoring. Several diseases such as asthma [8], bronchitis [9], heart disease [10], sleep apnea syndrome (SAS) [11], and pneumonia [12] can be monitored by the changes in respiratory rate and depth.

Nowadays, wearable electronics are more attractive for the real-time monitoring of human health and breath. To let the users feel comfortable when using the wearable breath monitor, it is important to develop sensors with ultrathin film and high flexibility. Moreover, continuous respiratory monitoring is one of the important parts to detect several types of heart and respiratory diseases [8,9,10,11,12]. Furthermore, breath analysis is also an important tool to detect various kinds of cancer such as that of the lung, breast, prostate, and colorectal [13,14,15,16]. To fulfill the fast-growing demand for wearable electronics in daily life for continuous respiratory monitoring, it is important to develop ultra-flexible, lightweight, and comfortable respiratory monitoring sensors. In recent years, there have been many flexible relative humidity sensors reported on substrates such as polyethylene terephthalate (PET/0.2–0.7 mm) [17,18,19,20,21], polyester (PE/0.1–0.5 mm) [20], polyimide (PI/0.125 mm) [21,22,23], polyethylene napthalate (PEN/50 µm) [21,24], poly carbonate (PC) [25], epoxy [21,26], cellulose paper [21,27,28], and textiles [29,30]. A comparison of these works with the key parameters (sensing material, substrate, deposition method, annealing temperature, dynamic range of sensor, and response and response/recovery time) is shown in Table S1 [17,18,19,22,23,24,27,28,30]. Specifically speaking, graphene/-Ag colloid film on PET substrate annealed by infrared light exhibits a response of 3.5% at 97% RH [17,21]. Graphene nanochannel-confined poly-dopamine (GNCP) film on PI substrate gives a 2,000,000% response under 90% RH [21,22]. Multiwalled carbon nanotube composite film via a poly-L-lysine modification (MWCNTs/PLL)-based humidity sensor on PI substrate at an annealing temperature of 60 °C shows a response of 659.97% at 91.5% RH [21,23]. SnS_2_/rGO, graphdiyne, and Cellulose NF/Carbon black-based humidity sensors printed on flexible substrates were also demonstrated [18,19,21,24]. In addition, ecofriendly cellulose paper is not only used as a flexible substrate but also as a humidity-sensing material [27,28]. The humidity sensor based on conventional printing paper together with flexible conductive adhesive tape shows a good response (~10^3^) in the range of 41.1 to 95.5% RH with good linearity. Moreover, the sensor shows a dynamic working range from 7.2 to 95.5% RH with a high response of ~350,000% [28]. The cellulose/KOH-based sensor shows a fast and reversible humidity response in the range of 11.2 to 97.7% RH [27]. These paper-based resistive sensors receive attention in respiratory monitoring applications [27,28]. Considering user convenience, integrating sensors directly on masks is also of great interest. Recently, a few sensors were developed on masks for continuous respiratory monitoring [21,22,30]. MXene/MWCNT-sensing material was dropped on MWCNT fabric (MC fabric) and cured by UV light. The electrical circuit was printed by conducting ink and encapsulated by polyimide to avoid short circuiting due to the water vapor. Finally, the whole sensor with the encapsulated circuit was attached inside the mask near the nose [30]. A GNCP-modified humidity sensor was also integrated into the oxygen supply mask for patients to continuously monitor their respiration by tracing the nasal breathing rate and depth [22]. In our work, we first demonstrated the use of food plastic wrap as the substrate material. With the ultra-flexible substrate, we can easily integrate the sensor onto the mask to detect the breathing pattern.

To adapt to the low-temperature resistance of plastic food packaging, we developed a low-temperature process based on NIR laser annealing to produce the SnO_2_-sensing layer. A literature survey reveals that metal oxide semiconductors (ZnO, SnO_2_, ITO, WO_3_, CuO, NiO) [31,32,33,34,35,36] are good candidates for humidity sensing. Metal oxide semiconductor (MOx)-based humidity sensors can be fabricated with a low-cost solution process to exhibit high sensitivity and a fast response [37]. Nevertheless, conventional metal oxide (MOx)-based sensors usually require a high annealing temperature (>200 °C) and high operating temperature (>100 °C) [38]. Recently, noble metals (Au/ZnO, Ag/SnO_2_) [35,39] and carbon-based nanomaterials (GO/ZnO, SnO_2_/rGO, g-C_3_N_4_/ZnO) [40,41,42] have been incorporated into metal oxides to realize room-temperature-operated MOx-based sensors. In Table S2, recently-published metal-oxide-based humidity sensors with a low annealing temperature (<100 °C) are listed [25,26,35,41,42,43,44]. It is noticed that, compared to composite MOx, pure MOx sensors show lower responses [26,35,42,43,44] under a low annealing temperature. The drop-casting or sputtered MOx/carbon nanomaterial composite-based humidity sensors such as In_2_O_3_/GO, SnO_2_/rGO, and g-C_3_N_4_/ZnO-based sensors on flexible epoxy and PI substrate are fabricated at a particularly low annealing temperature such as 60 °C [26,41,42]. As shown in Table S2, in our experiment, pure SnO_2_ was used as the sensing layer, and the low annealing temperature (<41 °C) was realized by using NIR laser annealing. Comparable sensing performance was achieved on the particularly soft plastic wrap substrates.

In this paper, we first evaluated the lyophilic nature (wettability property) of various food plastic wraps and pointed out the key to forming the sensing layer by spin-coating. Then, we developed a sol-gel-assisted SnO_2_ film on ultraflexible food plastic wrap (~10 µm) at low NIR (800 nm) annealing powers of 16–84 W/cm^2^ (1 min) with equivalent temperatures of 26.2–40.8 °C. Noted that the laser power was much lower than that used in our prior work, in which an NIR laser with powers of 93–157 W/cm^2^ for 1 min (equivalent temperatures of 110–228 °C) was used to treat the sol-gel IZO on PC substrate to serve as H_2_S gas sensors [45]. With the optimized condition, our proposed ultraflexible sensor exhibits a good response to RH from 15% to 70%. Most interestingly, the ultraflexible SnO_2_-based sensor can detect very small incremental changes (0.1% to 2.2%) of relative humidity in air. Finally, the proposed sensor was attached to a mask to show the feasibility of implementation. Real human nasal breath tests were also performed to show the ability to detect different breathing modes.

## 2. Experimental Procedure

### 2.1. Materials

Different kinds of plastic wrap such as HDPE/resin (Glad Press’n seal, Glad Products Company, Oakland, CA, USA), LDPE (Ming rong marketing Enterprises Ltd., Taipei, Taiwan), and PMP (Newtop. Com, Taipei, Taiwan) were purchased. PC substrates were purchased from General Silicon Co., Hsin-Chu City, Taiwan. Tin (iv) chloride pentahydrate (SnCl_4_, 5H_2_O) was purchased from Sigma Aldrich. All chemicals were used without any purification.

### 2.2. Sensor Fabrication

**Figure 1:**
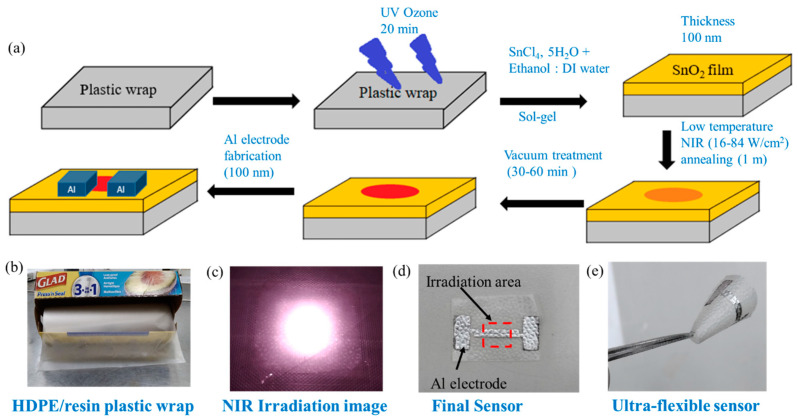
(**a**) Schematic diagram of SnO_2_-based sensor fabrication on HDPE/resin plastic wrap. Images of (**b**) HDPE/resin plastic wrap, (**c**) NIR irradiation on substrate, (**d**) SnO_2-_based sensor with Al electrode on plastic wrap, and (**e**) ultra-flexible sensor.

We dissolved 0.2 M SnCl_4_, 5H_2_O precursor in 1 ml of DI water and ethanol solution (1:1/4:1/1:3) under 24 h magnetic stirring to form a homogeneous sol-gel solution. The sensor was fabricated following a simple NIR annealing method (Figure 1a). Commercial HDPE/resin plastic wrap is shown in Figure 1b. Plastic wraps (HDPE/resin, LDPE, or PMP) was stuck to a 3 cm × 3 cm PC substrate. The plastic wrap substrates were kept under UV ozone treatment (Orient Service Co. Ltd.; TW-UN-URS-500-03) for 20 min to obtain a hydrophilic substrate. Then, 60 µL of the SnO_2_ precursor solution was spin-coated on plastic wrap with a spin rate of 800 rpm for 30 sec. After coating, the substrate was annealed under 808 nm NIR continuous laser (DS3-11312-xxx-LD No., BWT Beijing) irradiation for 1 min to form a SnO_2_ film. Since the irradiation area was only 5 × 5 mm^2^ (Figure 1c), not all of the substrate was dried. We then kept the substrate under vacuum treatment to evaporate the remaining solvent from the un-irradiated part for 30 min. The thickness of the SnO_2_ film (100 nm) was measured by ET200 (SANPANY INSTRUMENTS CO., LTD., Taipei, Taiwan) after annealing on a glass substrate. Finally, a 100 nm thick aluminum electrode was deposited using thermal evaporation under a high vacuum condition (*p* < 4 × 10^−6^ Pa) (Figure 1d). The distance (i.e., channel length) between two electrodes was 200 µm, and the channel width was about 1.2 cm. We have prepared a flexible gas sensor, which is shown in Figure 1e. In later sections, we compare devices with four annealing conditions such as an only-vacuum-annealed device (noted as S-vac) and devices with 1 min NIR laser annealing at 16, 33, and 84 W/cm^2^ (noted as S-16, S-33, and S-84) (Table S3). Sensors on glass substrates using thermal annealing at 200 °C were used as reference devices.

### 2.3. Sensor Measurement System 

The relative-humidity-sensing measurement system included a syringe pump system, sensing glass chamber, commercial relative humidity meter, mass flow meters, I-V analyzer (Keithley 2400), and real-time measurement system (Keysight U2722A) (Figure S1) [45,46]. The response was measured by the ratio of change in the current (µI) to initial current (*I_initial_*). The response is calculated using (Equation (1)) below.

(1) $$\mathrm{Response}=\frac{\Delta I}{I_{initial}}\times 100$$

First, the relative humidity of the sensing chamber must be controlled by the mass flow meters of “dry air” (directly from the air cylinder) and “wet air” (through the water chamber). We modulated the relative humidity to 15%, 20%, 30%, 40%, 50%, 60%, and 70%, and the relative humidity was monitored by a commercial relative humidity sensor (CHY-321 Thermo Hygrometer). After placing the flexible sensor inside the sensing chamber, we needed to wait for a stable electrical signal in real-time measurement using Keysight U2722A at a fixed voltage of 7 V. The current was traced when tuning the RH levels. The flow rate of the total gas flow was fixed at 500 mL/min. To also place an incremental change in the RH level, the syringe pump was used to inject pure nitrogen into the sensing chamber. Tuning the syringe pump can cause a 0.1% to 2.2% RH change in the chamber. When testing the selectivity, the syringe pump then injected various analytes such as NO, NO_2_, acetone, and NH_3_ under a fixed background relative humidity (RH 60%). The concentration of the analyte gas was controlled by tuning the flow rate and volume of the syringe pump system. Moreover, the injection time was fixed to be 30 s in the syringe pump system. All the measurements were carried out at room temperature (24 °C), which was controlled by the air conditioner.

## 3. Results and Discussions

### 3.1. Wettability Effect and NIR Laser Endurance

**Figure 2:**
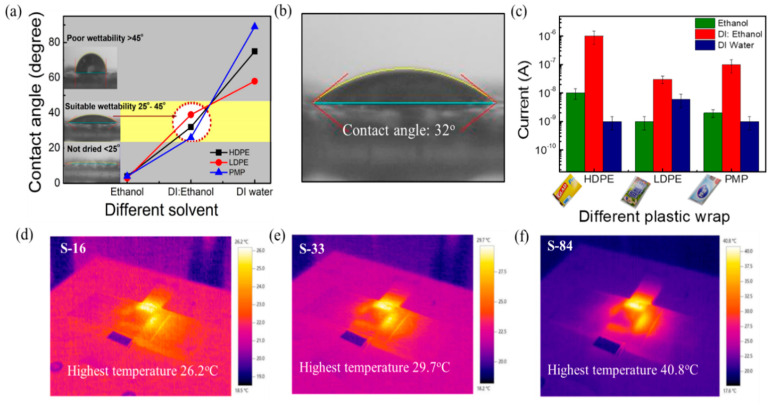
Selection of plastic wrap and solvent. (**a**) Measurement of contact angle for wettability test on different plastic wraps (HDPE/resin, LDPE, and PMP) using different solvents (ethanol, DI water: ethanol, and DI water). (**b**) Image of contact angle using DI water: Ethanol (1:1) solution on HDPE/resin plastic wrap. (**c**) Current of the film of different plastic wraps using different solutions. Thermal images of (**d**) **S**−**16**, (**e**) **S**−**33**, and (**f**) **S**−**84**.

When using the solution process to form the semiconductor film, the weak affinity between the substrate and solvent leads to poor thin film uniformity and hence poor device electrical properties and stability. Thus, the exploration of how to find a proper solvent to form a uniform sensing film on various kinds of plastic wraps is important. The hydrophilicity of the plastic wrap substrates was tested by contact angle measurement (FTA125, First Ten Angstroms USA). The contact angles of solvents on different plastic wraps are shown in Figure 2a. It is noticed that the contact angles of DI water on HDPE/resin, LDPE, and PMP plastic wraps are 75°, 58°, and 89°, respectively. When introducing the spin-coating process, a solvent such as DI water spills out without staying on the substrate. On the other hand, the contact angles of ethanol on HDPE/resin, LDPE, and PMP plastic wraps are 4°, 3°, and 4°, respectively. The plastics exhibit good wettability in ethanol (contact angle of < 5°). However, the remaining solvent in the thin film is too much and is difficult to be removed after spinning (800 rpm, 30 s) to produce a dried film. As a result, we mixed DI water and ethanol at various ratios to find the condition with suitable wettability and proper drying. The optimal contact angle between the mixed solvent and the plastic wraps was found to be about 25° to 45°. Specifically speaking, as shown in Figure 2a, the optimal DI water to ethanol ratio is 1:1 for HDPE/resin, 4:1 for LDPE, and 1:3 for PMP, while the corresponding contact angle is 32° (Figure 2b), 39°, and 26°, respectively. In Figure 2c, we also compared the obtained current (I) of the SnO_2_ film when using the various solvents (pure DI water, pure ethanol, and the mixed solution) on the three kinds of plastic wrap substrates. The mixing ratios were mentioned above. It is observed that SnO_2_ on HDPE/resin exhibited the highest current (5 × 10^−7^ to 4 × 10^−6^ µA). The current is higher than LDPE (~10^−8^ µA) and PMP (7 × 10^−8^ to 2 × 10^−7^ µA) under a 7 V biasing voltage at 60% RH. It is also noticed that LDPE and PMP substrates took a longer time (~1 h) to dry the film under vacuum. Spin-coating the sol-gel mixture on the HDPE/resin substrate can lead to a dry film within 30 min under vacuum. Note that the commercially available HDPE/resin plastic wrap exhibits roughly 0.01 mm textures on the surface. In the following experiments, we selected the HDPE/resin plastic wrap as the substrate and used the DI water: ethanol (1:1) mixed solution in the sol-gel solution process. 

To ensure not to damage the substrate, it is also essential to understand the substrate temperature under NIR laser irradiation. A thermal imager (Testo 875i-versatile) was used to understand the local thermal heating of NIR laser annealing of SnO_2_-covered substrates. The real-time thermal images captured under NIR irradiation for 1 min with powers of 16, 33, and 84 W/cm^2^ (noted as **S**−**16**, **S**−**33**, and **S**−**84**) are shown in Figure 2d−f, respectively. The images revealed that the maximum generated temperatures on the substrates were 26.2 °C, 29.7 °C, and 40.8 °C for **S**−**16**, **S**−**33**, and **S**−**84**, respectively. In the following experiments, the NIR laser annealing conditions (**S**−**16**, **S**−**33**, and **S**−**84**) did not cause a substrate temperature higher than 41 °C. In Table S3, it is obvious that the endured temperature for plastic wrap is low, and the 1 min NIR laser annealing method plays an essential role for the proposed sensors.

### 3.2. Material Analysis

**Figure 3:**
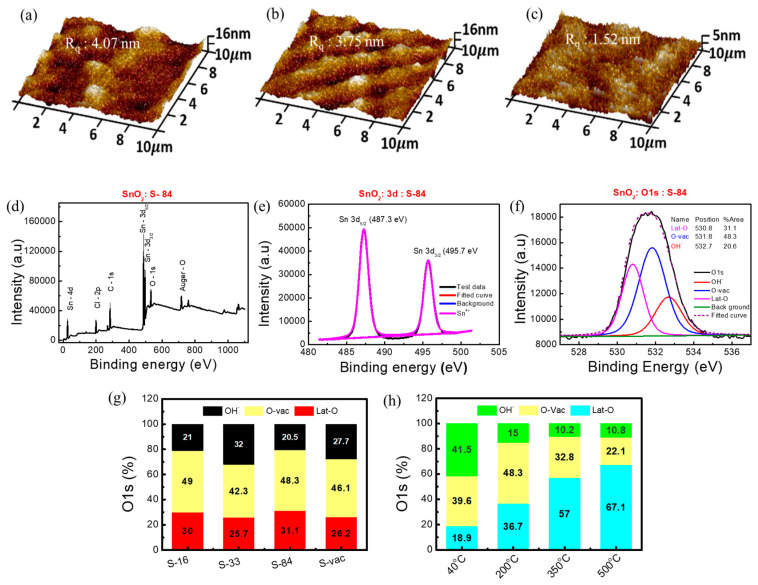
AFM images of (**a**) **S**−**16**, (**b**) **S**−**33**, and (**c**) **S**−**84**. XPS spectra of (**d**) SnO_2_ film grown on HDPE/resin plastic wrap, (**e**) Sn 3d, (**f**) O1s with three components, Lat-O, O-vac, and OH^−^. Analysis O1s data of (**g**) SnO_2_ on HDPE/resin substrate and (**h**) SnO_2_ on glass substrate.

Atomic force microscopy (AFM, Bruker) analysis has been used to study the morphology of the HDPE/resin substrate before and after different NIR irradiations. Additionally, the morphology of the SnO_2_ film on the HDPE/resin substrate was analyzed. The AFM image of only the HDPE/resin plastic wrap before NIR laser treatment showed a surface roughness (R_q_, rms value) of 3.63 nm in Figure S2a. After NIR laser irradiation, the surface roughness of only HDPE/resin slightly decreased from 3.25 nm to 2.96 nm with increasing NIR power from 16 W/cm^2^ to 84 W/cm^2^ (Figure S2b,c). The surface roughness of the SnO_2_ film on the HDPE/resin substrate also decreased from 4.07 nm to 3.75 nm then to 1.52 nm when increasing laser power from 16 W/cm^2^ to 33 W/cm^2^ then to 84 W/cm^2^ (Figure 3a–c). On the other hand, the vacuum-treated SnO_2_ film (**S-vac**) showed an increased surface roughness of 6.29 nm (Figure S2d). This result indicates that NIR annealing does not cause pore generation and may improve surface smoothness. Moreover, the surface roughness of **S**−**84** (1.52 nm) is comparable to that of the thermal-annealed sample with 200 °C 1 h annealing (1.22 nm) (Figure S2e). The SnO_2_ film annealed at a low temperature in our experiment may be an amorphous thin film. According to [47], coating the tin chloride precursor (dissolved in ethanol) at room temperature was reported to form an amorphous film, whose XRD data did not show any obvious peak corresponding to crystal planes.

To confirm the formation of metal oxide bonding under a low annealing condition, we implemented XPS analysis on the proposed SnO_2_ samples on HDPE/resin. A literature survey states that the presence of rich oxygen vacancy in SnO_2_ metal oxide plays a crucial role in humidity sensors [48]. Thus, XPS analysis was utilized to understand the formation of SnO_2_ metal oxide after NIR laser annealing as well as conventional thermal annealing (as control samples). XPS analysis on pure plastic wrap substrates was also conducted. PHI Quantera II (ULVAC) was used for XPS measurement, and Casca XPS was used to analyze XPS data. All data were calibrated using a carbon binding energy of 284.6 eV. The XPS spectrum of **S**−**84** is shown in Figure 3d. The presence of chloride (Cl) is due to the precursor SnCl_4_, 5H_2_O. The appearance of the C-1s peak was attributed to the possible contamination of organic residue after the deposition of the film. Sn-3d and O-1s peaks relate to SnO_2_ film formation after annealing. For further information, we analyzed the XPS spectrum for three kinds of plastic wraps after and before applying an NIR laser power of 84 W/cm^2^ (1 min) without any film formation. It is clearly noticed that the substrates only contain C-1s and O-1s elements (Figure S3a–c). We confirmed that Sn-3d, Cl-2p, and Sn-4d peaks are only observed after forming the SnO_2_ film on the substrate. The 3d peak of Sn contains two doublet peaks of 3d_5/2_ and 3d_3/2_ at 487.3 eV and 495.8 eV, respectively, in **S**−**84** (Figure 3e). These are the signature peaks of Sn^4+^ in SnO_2_ [49,50]. These two peaks are originated from spin orbit-splitting. Moreover, Table S4 shows the percentage contribution of C-1s, O-1s, and Sn-3d. The relative concentration “n” was calculated by the percentage concentration ratio of O-1s and Sn-3d (i.e, [O]/[Sn]). The calculated “*n*” value of the vacuum and NIR-annealed film is 2.19 ± 0.4. This “*n*” value is equivalent to that (*n* = 2.12) of the film annealed at 200 °C (Table S4). These results clearly prove that the material is SnO_2_ after laser curing. As expected, O-1s contains three components—metal oxide lattice oxygen (Lat-O) at 530.8 ± 0.1 eV, oxygen vacancy (O-vac) at 531.75 ± 0.15 eV, and a hydroxyl group (OH^−^) at 532.65 ± 0.15 eV (Figure 3f) [50]. In Figure 3g, the percentage ratios between Lat-O, O-vac, and OH^−^ in SnO_2_ samples with **S**−**16**, **S**−**33**, **S**−**84**, and **S**−**vac** are shown for comparison. All raw XPS data are shown in Figures S3 and S4. The SnO_2_ samples with **S**−**16** and **S**−**84** have comparatively more oxygen vacancy defects (O-vac) and metal oxide lattice oxygen (Lat-O) than the SnO_2_ samples with **S**−**33** and **S**−**vac** [Figure 3g and Figure S3d–f]. In Figure 3h, the thermal-annealed SnO_2_ samples on glass substrates with four different annealing temperatures (40 °C, 200 °C, 350 °C, and 500 °C) for 1 h were also analyzed as a comparison. The percentage area ratios of Lat-O and O-vac in SnO_2_ with **S**−**16** and **S**−**84** are much higher than those in 40 °C 1 h samples and are comparable with those in 200 °C 1 h samples. This result indicated that the NIR laser energy can be effectively transformed into local energy to help the formation of metal–oxygen networks in the sol-gel process. The 1 min laser irradiation can be used to form a useful M-O semiconductor film while the local temperature can be kept low (40 °C). The high level of O-vac in SnO_2_ with **S**−**16** and **S**−**84** may also facilitate relative humidity sensing. According to the literature, in the M-O semiconductor, the presence of rich surface oxygen vacancy defects stimulates the absorption of water molecules on the surface and improves the response of sensors. Moreover, it also accelerates the dissociation of water molecules into H_3_O^+^ or H^+^ [48]. In the following section, we will investigate the relative-humidity-sensing response in the proposed sensors. 

### 3.3. Relative Humidity Response Measurement

**Figure 4:**
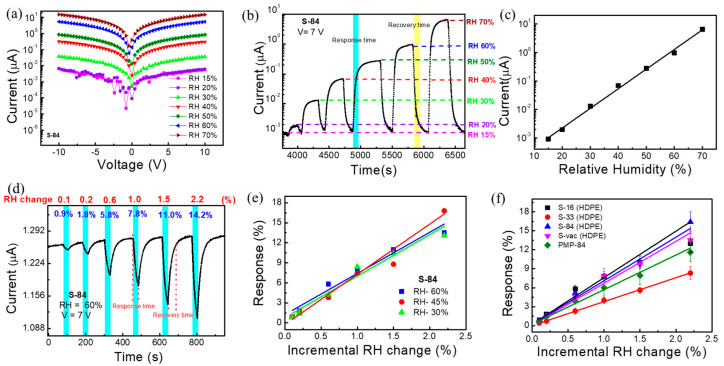
(**a**) I-V characteristic plot of SnO_2_-based sensor **S**−**84** under different relative humidities. (**b**) Current–time measurement curve of **S**−**84** under different relative humidities. (**c**) Calibration curve of relative humidity response under different RH. (**d**) The incremental change response under 60% RH. (**e**) Incremental change response vs. low-level RH change data plot under different relative humidities of **S**−**84**. (**f**) Calibration curve of incremental change response under low-level RH change for different samples **S**−**16**, **S**−**33**, **S-84, S**−**vac**, and **PMP**−**84**.

In this section, we will discuss relative humidity sensing responses in two ways, such as the **wide-dynamic-range response** and the detection of the **incremental change response.** The current voltage (I-V) characteristic curves of **S**−**84** under different relative humidities are shown in Figure 4a. It is clearly observed that the current level increases with increasing relative humidity. This kind of relative-humidity-enhanced current conduction is known to be caused by the dissociation effect of absorbed water molecules on the surface of MOx molecules [45]. The XPS result of **S**−**84** may show how the oxygen vacancy promotes the formation of conductive ions such as H+ and H_3_O^+^. This increases the current through the hopping of these ions between neighboring hydroxyl groups [48]. According to the literature, the surface with hydroxyl functional groups can provide a hopping path for proton transport. Water molecules may cover such surfaces to promote hopping [51]. In our low-temperature SnOx layer, the amorphous SnO_2_ may be mixed with some remaining byproducts (such as hydroxides, SnOH, and contamination of organic residue). Hence, the electron-conducting path may be formed by interconnecting the high-level oxygen vacancy network as well as the hydroxyl-rich parts. Hence, in a high relative humidity environment, water molecules cover the hydroxyl-rich parts to promote the hopping-related current. It is plausible that the very sensitive RH response is because the hopping serves as a bottleneck in the conducting path. Thus, the surface current level increases after attaching water molecules to the SnO_2_ film. To keep a high enough current level when tuning the relative humidity, we chose 7 V to bias the sensors in the following sensing response measurement. 

The **wide-dynamic-range responses to** relative humidity, RH (%), of 20%, 30%, 40%, 50%, 60%, and 70% are plotted in the current vs. time plot in Figure 4b. The measurement system is shown in Figure S1, and the background air exhibited a relative humidity of 15%. As mentioned before, increasing the relative humidity increases the current level. The response time, Rs_T90_, (marked in cyan blue color) and the recovery time, Rc_T90_, (marked in yellow color) of the proposed sensor are 90 s and 150 s, respectively. Here, T90 indicates the time taken by the sensor to show 90% of its initial response (Figure 4b). It is noted that, in a later section when performing real-time breath monitoring, we do not need to wait for saturation. As compared in Table S2, the response time (90 s) is not particularly low but is comparable to most prior reports. The sensing response was defined as the current change amount divided by the current in background air (1 nA in RH:15%) and is plotted as a function of relative humidity in Figure 4c. Specifically, when the relative humidity changed from 15% to 70%, the response was as high as 324,000% for **S**−**84**. The linear line obtained in Figure 4c indicated a power law dependence between the current conduction and the relative humidity, which agrees with prior reports [52]. It is noted that the detected range (RH 15% to 70%) was limited by our relative humidity control system. The linear curve in Figure 4c implied a wider detective range of the proposed sensor. 

In some applications, it is also needed to detect a very small change in relative humidity. Hence, we wanted to investigate the incremental change (0.1~2.2% change) in relative humidity. The response is named the **incremental change response**. As shown in Figure 4d, the background relative humidity was fixed at 60%, and then an incremental change was applied to reduce RH to 59.9, 59.8, 59.4, 59.0, 58.5, and 57.8%, i.e., the change in RH was 0.1, 0.2, 0.6, 1, 1.5, and 2.2%, respectively. To realize this measurement, the sensing system in Figure S1 was used while dry nitrogen air was injected by a syringe pump into the gas flow and the flow rate was fixed at 500 mL/min. A commercial bulky and expensive RH sensor was also placed to simultaneously detect the RH level as a reference. As shown in Figure 4d, the sensing period was 30 secs (marked by cyan blue) when injecting pure nitrogen by using a syringe pump. The recovery period was 29 to 50 s when the background air with an RH of 60% was continuously purged. The **incremental change response**, defined as the current change amount during the 30 sec sensing period divided by the initial current level, of **S**−**84** was noted as 0.9%, 1.8%, 5.8%, 7.8%, 11%, and 14.2% in Figure 4d. After only slightly changing the value of RH (0.1–2.2%), the current dropped very fast during the response time of 30 s, whereas after injecting 60% RH in the recovery period, the current reached its initial position, showing a recovery time of 29 s after a 0.1% change and 50 s after a 2.2% change. The sensing and the recovery are both quite fast. More interestingly, when plotting **incremental change responses** as a function of the RH change in Figure 4e with various background RHs, almost the same **incremental change responses** were obtained when changing the background relative humidity from 30% to 45% and to 60%. Specifically, with 1% RH changes when changing the relative humidity from 60% to 59%, 45% to 44%, and 30% to 39%, the responses were 7.8%, 7.4%, and 8.4%, respectively (Figure S5a). Thus, these results reveal that incremental RH changes cause a consistent current variation ratio even with different current levels. 

The effect of different NIR annealing powers on the RH response of the sensors was investigated. The **incremental change responses** as a function of the RH change (i.e., calibration curves) are plotted in Figure 4f for **S**−**16**, **S**−**33**, **S**−**84**, **S**−**vac**, and **PMP**−**84** sensors. It shows that the responses are quite similar for **S**−**16**, **S**−**84**, and **S-vac**, whereas the **S-33** and **PMP**−**84** sensors exhibit comparatively low responses (Figure 4f). The I-V characteristic plot of different samples is shown in Figure S5b, which indicates that the current level of all samples varied from 10^−6^ to 10^−7^ A at 7 V. Considering their XPS analysis results in Figure 3g, it is plausible that the similar responses of **S**−**16**, **S**−**84**, and **S**−**vac** are because of the presence of similar amounts of oxygen vacancy on the surface, resulting in adsorption of about the same amount of water molecules on the surface. For **S**−**33**, the particularly low level of oxygen vacancy may deteriorate the absorption of water molecules and hence the sensing response. It is also worth noting that, in addition to the HDPE/resin substrate, we also repeat the measurement of **incremental change responses** for SnO_2_ sensors on PMP food plastic wrap. The NIR laser treatment condition is also 84 W/cm^2^ (1 min), so the sample was named **PMP**−**84**. The I-V characteristic and the real-time **incremental-change responses** are shown in Figure S5c,d. It also exhibits 0.7, 1.4, 3.9, 6.0, 7.9, and 11.6% responses under 0.1, 0.2, 0.6, 1, 1.5, and 2.2% relative humidity changes, respectively. These results reveal that the proposed NIR-laser-treated SnO_2_ sensors can be formed successfully on different plastic wrap substrates and a deliver very sensitive response to a relative humidity change of only 0.1%. 

### 3.4. Selectivity, Flexibility, and Reliability

**Figure 5:**
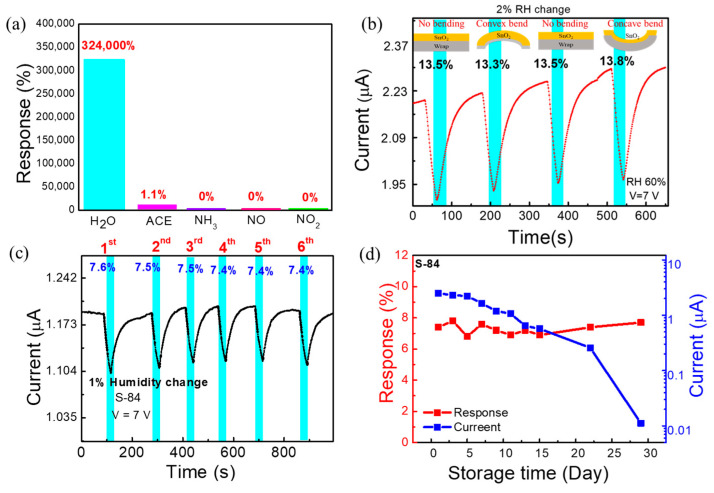
(**a**) Selectivity test under 1 ppm of different gases compared with 15–70% RH (H_2_O) (**b**) The real-time measurement of sensor at 2% RH change under different bending conditions (convex, concave, and no bending). (**c**) Repeatability test under 1% RH change. (**d**) Response and current data of **S**−**84** during 29 days of storage time.

Selectivity tests to different gas molecules were carried out to check the potential of our sensors for selective detection of the moisture level in breath. It is reported that there are many other compounds, in amounts of parts per trillion (ppt) to parts per million (ppm), found in the highly humid human exhaled breath [21]. Thus, the sensor was tested for different gas species at 1 ppm concentration. A graph depicting responses to different gas molecules at 1 ppm is shown in Figure 5a. The **S**−**84** sensor exhibited high selectivity towards H_2_O molecules. Specifically, no response to 1 ppm NH_3_, NO, or NO_2_ molecules was recorded. Only a small response (1.1%) to 1 ppm acetone was observed. To understand the performance of the flexible humidity sensor at different bending conditions, the response of the sensor was measured at the natural flat and bending conditions (convex and concave) under 60% RH with 2% incremental RH change (Figure 5b). The measured responses are 13.5, 13.3, and 13.8% for flat, convex, and concave shapes of the substrate, respectively. The flexibility test shows that the response is almost unchanged under different bending conditions. 

To enable practical applications, we also evaluated reliability tests including the repeatability and the storage lifetime measurement. The repeated response data of **S**−**84** under 1% RH change is shown in the current vs. time plot in Figure 5c. The 1% RH change (60% to 59% then returned to 60%) was repeated in six cycles, and the response was 7.5 ± 0.1 %. The sensing time was 30 secs, and the recovery time was at least 48 s. This illustrates good repeatability of the response. The storage lifetime test (response vs. storage time plot) was executed by measuring the response of the sensor under 1% RH (60%−59%) change in a 2-day interval up to 15 days and in a 7-day interval up to 29 days (Figure 5d). The measurement was performed up to 29 days. Day 1 is indicated as the next day of sensor fabrication. After device fabrication, the sensor was stored in ambient air. The response (7.3 ± 0.4%) of the sensor showed negligible changes between day 1 to day 29. The current vs. storage time data are also shown in Figure 5d. There was approximately a 5% degradation of the current level every 2 days up to day 15. Within about 2 weeks, the sensor current could be kept at the µA current level. Then, from day 15 to day 29, it decayed rapidly. The mechanism to explain the degradation of the current level with storage days is still not investigated. However, regardless of the current change, the responses remained almost the same after 29 days (Figure 5d). Considering that the sensors were just simply kept in ambient atmosphere without any additional preservation, the good repeatability and the good-enough lifetime make the proposed sensors promising for future applications. In the following section, we used the **S**−**84** sensors to perform real respiration detection.

### 3.5. Respiratory Sensing Performance

**Figure 6:**
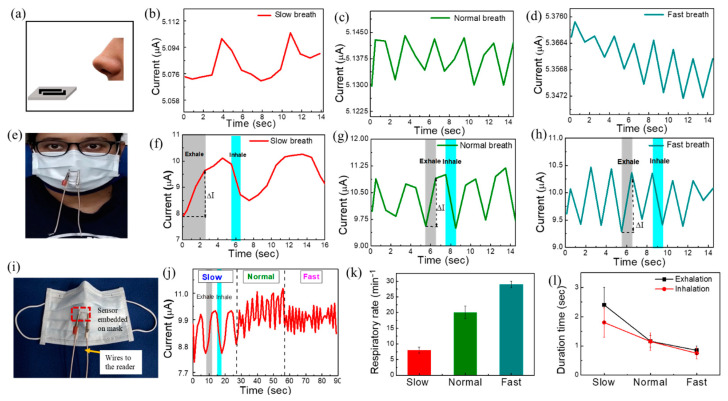
(**a**) Sensor was placed 5 cm away from nose under ambient atmosphere without any additional flow control or sensing chamber setup. Human breath response to (**b**) slow, (**c**) normal, and (**d**) fast breathing under ambient atmosphere. (**e**) The image of human wearing sensor-embedded mask. Human breath responses curve to (**f**) slow, (**g**) normal, and (**h**) fast breathing after wearing mask (sensor 1 cm far from nose). (**i**) Image of ultra-flexible sensor embedded on smart mask. (**j**) The data of continuous monitoring of breath with different breathing modes. (**k**) The respiratory rate data for slow, normal, and fast breath. (**l**) Duration time of exhalation and inhalation.

In this section, we used the proposed **S**−**84** sensors to detect respiratory patterns. The study protocol was approved by the institutional review board (no. 202101239B0C502). Slow, normal, and fast respiratory patterns were monitored by the real-time current curves given in Figure S6. The **S**−**84** sensor was put inside a glass chamber with a fixed background relative humidity of 70%, 60%, and 50%. Continuous slow, normal, and fast nasal breath was released 5 cm away from the substrates (Figure S6a). Noted that the respiratory rate for slow, normal, and fast breath are 8 ± 1, 20 ± 2, and 29 ± 1 per minute, respectively. Similar conditions were used in prior reports [21,53]. It is noticed that the current increases during exhaled breath (indicated by arrows) with an amount (ΔI) of about 0.02 ± 0.01 µA, and then it returns to its original position in inhalation (Figure S6b–j). The results reveal that the sensor is capable of tracing continuous respiratory patterns at different speeds (7 to 30 times per minute) and various background RH levels. Then, we further placed the sensor 5 cm away from the nose under ambient atmosphere without any additional flow control or sensing chamber setup (Figure 6a). The open-air relative humidity was 70% measured by a commercial relative humidity meter. Our proposed sensor also can detect slow, normal, and fast exhale breathing from the human nose (Figure 6b–d). The respiratory detection results under chamber at 70% RH (Figure S6b–d) and outside of the chamber in ambient atmosphere (70% relative humidity measured by commercial relative humidity meter) (Figure 6b–d) are unchanged. The average values of ΔI about 0.03, 0.015, and 0.008 µA for slow, normal, and fast breathing for both cases, respectively. According to the literature, the respiratory rate is 12–20 breaths per minute (bpm) for a healthy adult person, and it shows abnormality in the range of <6 or >24 bpm [53]. Our proposed sensor shows a normal respiratory rate of 20 ± 2 bpm and abnormal respiratory rate (after exercise) of 29 ± 1 (>24) bpm, which is comparable to the literature. Respiratory diseases such as the slow breathing disease of sleep apnea cause breathing stops for 10 to 20 s, while asthma causes fast breathing (>30 breaths/min) [8,11]. There is no adverse event noted while using our sensor for the test subjects (*n* = 2).

To show the advantage of easy integration with masks, we attached the **S**−**84** sensor to a conventional face mask for continuous respiratory monitoring (Figure 6e). Figure 6i shows the smart mask image with the embedded flexible sensor. To confirm the worst conditions when the breath was filtered by the face mask, we intentionally placed the sensor 1 cm ahead of the mask. Then, the sensor was performing measurements when testers wore face masks and exhaled/inhaled in slow, normal, and fast breathing conditions [Figure 6f–h]. After wearing the face mask, the relative humidity in front of the face mask was about 75–85%, which was higher than the ambient RH of 70%. As a result, the current level in sensor **S**−**84** increases to about 10 µA. The net changes in current (ΔI) are 1.62, 1.34, and 1.03 µA during exhalation/inhalation for slow, normal, and fast breath, respectively. Noted that ΔI can be enlarged by introducing comb-like electrodes in future works. The corresponding gray and cyan-blue rectangles in Figure 6f–h represent the time of exhalation and inhalation. The continuous monitoring of breath (current vs. time) shows the gradual decrease in ΔI in slow to fast breathing modes (Figure 6j). ΔI increases with slow (deep) breathing due to the large intake of air volume, i.e., the large number of interacting molecules present in the air could interact with the sensor as compared to fast breathing. The respiratory rate was counted manually by noticing the peaks obtained from this continuous monitoring plot. As mentioned above, the respiratory rate for slow, normal, and fast breath are 8 ± 1, 20 ± 2, and 29 ± 1 per minute, respectively (Figure 6k). The duration time is determined by observing how long the current change (ΔI) is upon one exhale or inhale breath. It was noticed that the periods of exhalation are 2.4 ± 0.6 s, 1.16 ± 0.2 s, and 0.85 ± 0.15 s, while those of inhalation are 1.8 ± 0.5 s, 1.15 ± 0.3 s, and 0.75 ± 0.2 s for slow, normal, and fast modes of breath, respectively (Figure 6l). The clear sensing responses confirm that the proposed ultra-flexible breath sensors are suitable for detecting respiration patterns even with a face mask. With the ultra-flexible plastic wrap substrate, the sensor can be easily integrated with other objects with arbitrary shapes. 

## 4. Conclusions

The ultrathin and ultra-flexible relative humidity sensor with SnO_2_ thin film on plastic wrap was successfully demonstrated by NIR laser annealing at low powers (16–84 W/cm^2^) at room temperature. To obtain a uniform sol-gel SnO_2_ thin film, the suitable wetting angle on the plastic wrap needs to be controlled between 25 and 45°. The thermal imager reveals that the equivalent temperature is as low as 26.2–40.8 °C, corresponding to the 1 min irradiated NIR laser power of 16–84 W/cm^2^. Moreover, the XPS results prove the presence of high-level oxygen vacancies on the surface of the sensor, which may facilitate the absorption of water molecules and allow a fast-enough response and recovery time (<30 s). This sensor exhibits a wide dynamic range to detect relative humidity from 15% to 70%, and it also has high sensitivity to detect 0.1% to 2.2% incremental changes in relative humidity. With the ultra-flexible substrate, the sensor can be easily attached onto a face mask. When placing the proposed sensor in front of the human nose covered by a mask, the sensor can clearly reflect the respiration pattern in slow, normal, and fast breathing. Thus, this sensor can be a good candidate in the field of respiratory disease monitoring. The proposed “sensor on mask” technology may be a comfortable and convenient approach for continuous breath detection.

## Data Availability

Data will be made available on request.

## Supplementary Materials

The following supporting information can be downloaded at: https://www.mdpi.com/article/10.3390/bios13010081/s1, Figure S1: Diagram of sensor measurement system; Figure S2: AFM images of (a) Only HDPE/resin. (b) HDPE/resin under NIR laser power 16 W/cm^2^ and (c) 84 W/cm^2^. (d) HDPE/resin/SnO_2_: S−vac (e) Glass/SnO_2_ under thermal annealing at 200 °C; Figure S3**:** XPS data of (a) HDPE/resin, (b) LDPE, (c) PMP before (only substrate, substrate with NIR irradiation 84 W/cm^2^) and after depositing film (SnO_2_ film with NIR irradiation 84 W/cm^2^). XPS O-1s for (d) S−33, (e) S−84 and (f) S−vac; Figure S4**:** SnO_2_: O-1s data of thermally annealed film at (a) 40 °C, (b) 200 °C, (c) 350 °C, and (d) 500 °C; Figure S5: (a) Real time measurement of 1% RH changes under different relative humidity (b) I-V of different sensors (S−16, S−33, S−84, and S−vac) under 60% RH (c, d) I-V and incremental-change of humidity response of PMP-84 under 0.1% to 2.2% RH; Figure S6: (a) Image of breath analysis inside glass chamber. The continuous slow, normal, and fast nasal breath was released from 5 cm away from substrates under different background relative humidity environment such as; (b-d) 70% RH (e-g) 60% RH, and (h-j) 50%; Table S1: Recent flexible humidity sensors; Table S2: Recent metal oxide based humidity sensors under low curing temperature; Table S3: Fabrication of plastic wrap sensor under different annealing condition.
