# Supplementary material for: SnO2-Based Ultra-Flexible Humidity/Respiratory Sensor for Analysis of Human Breath
Source: Biosensors (Basel). 2023 Jan 3;13(1):81. doi: 10.3390/bios13010081 (PMC9856198; doi:10.3390/bios13010081)
Supplement: Supplementary file 1 [file biosensors-13-00081-s001.zip › biosensors-2099938-supplementary.pdf]

biosensors

Supporting Information

SnO_2-Based Ultra-flexible Humidity/Respiratory Sensor for Analysis of Human Breath

Moumita Deb [1,2], Mei-Yu Chen [3], Po-Yi Chang [1,2,4,5], Pin-Hsuan Li [1,2], Ming-Jen Chan [6,7,8], Ya-Chung Tian [6,7], Ping-Hung Yeh [3], Olivier Soppera [4,5,*] and Hsiao-Wen Zan [1,2,*]

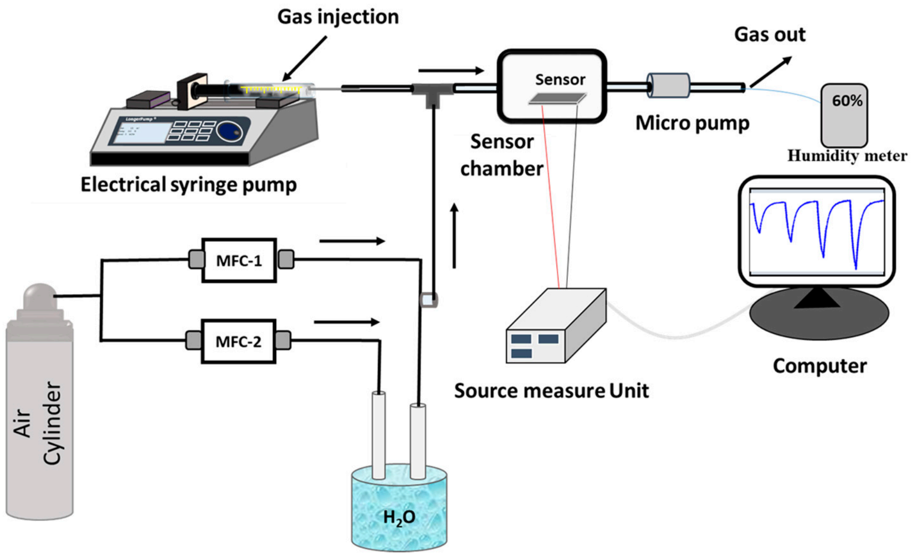

Figure S1. Diagram of sensor measurement system.

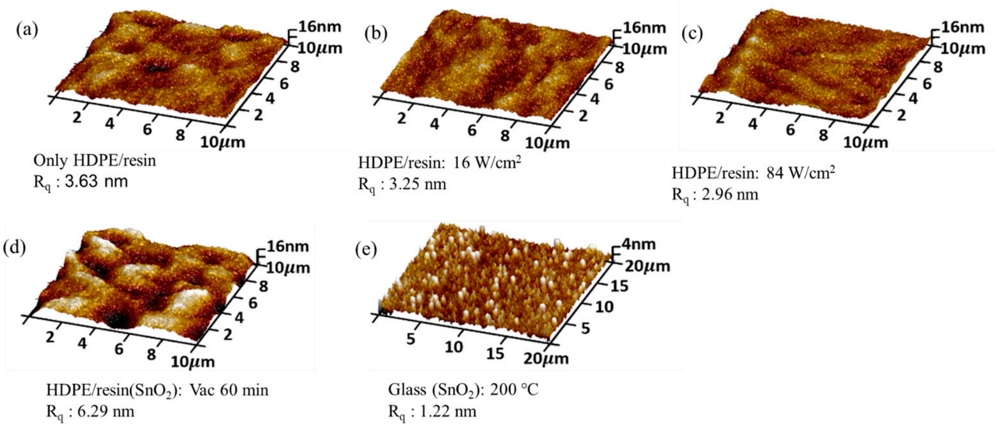

Figure S2. AFM images of (a) Only HDPE/resin. (b) HDPE/resin under NIR laser power 16 W/cm² and (c) 84 W/cm². (d) HDPE/resin /SnO_2: **S−vac** (e) Glass/SnO_2 under thermal annealing at 200 °C.

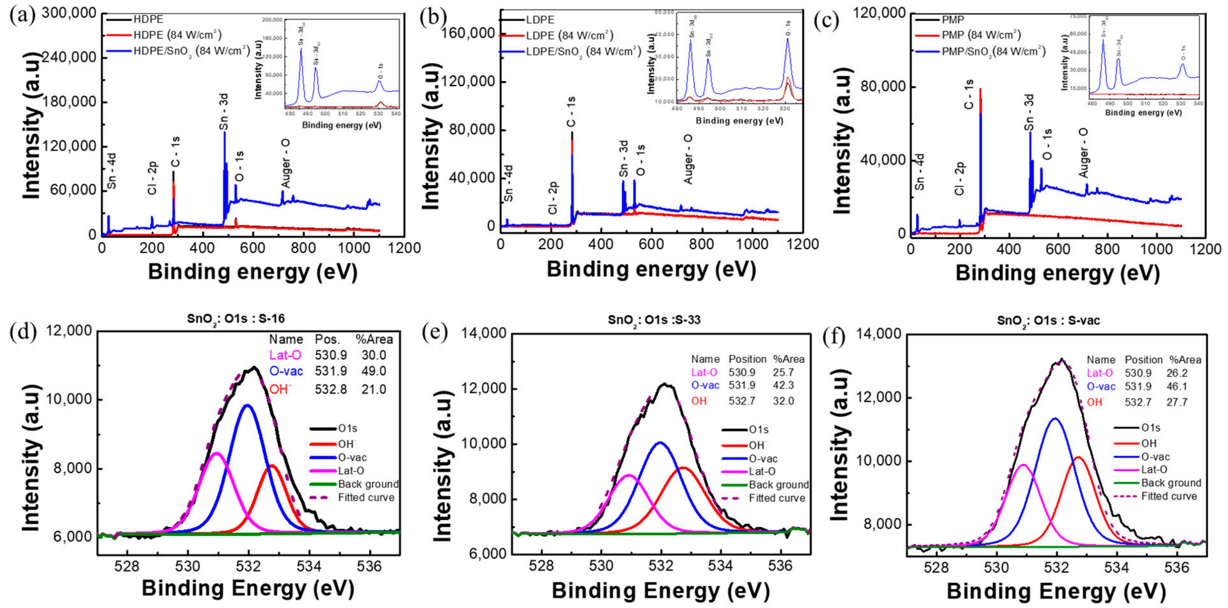

Figure S3. XPS data of (a) HDPE/resin, (b) LDPE, (c) PMP before (only substrate, substrate with NIR irradiation 84 W/cm²) and after depositing film (SnO_2 film with NIR irradiation 84 W/cm²). XPS O-1s for (d) **S−33**, (e) **S−84** and (f) **S−vac**.

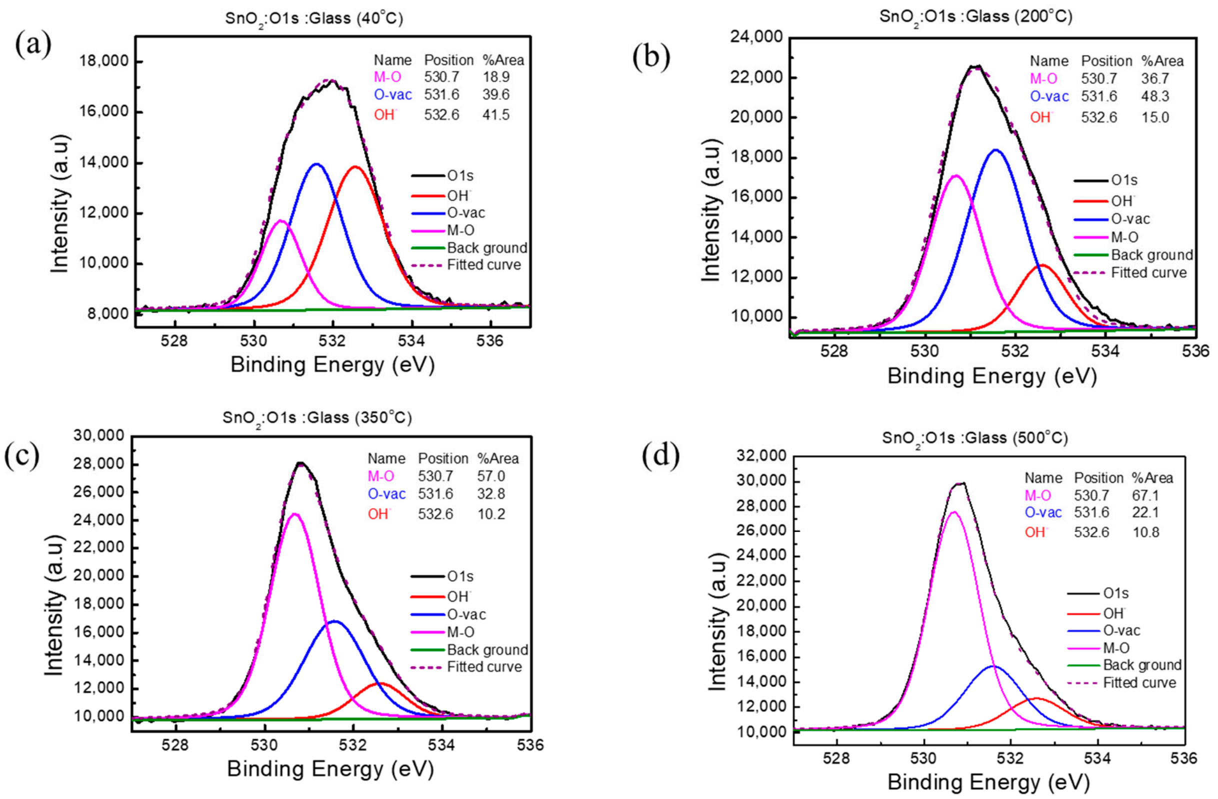

Figure S4. SnO_2: O-1s data of thermally annealed film at (a) 40 °C, (b) 200 °C, (c) 350 °C, and (d) 500 °C.

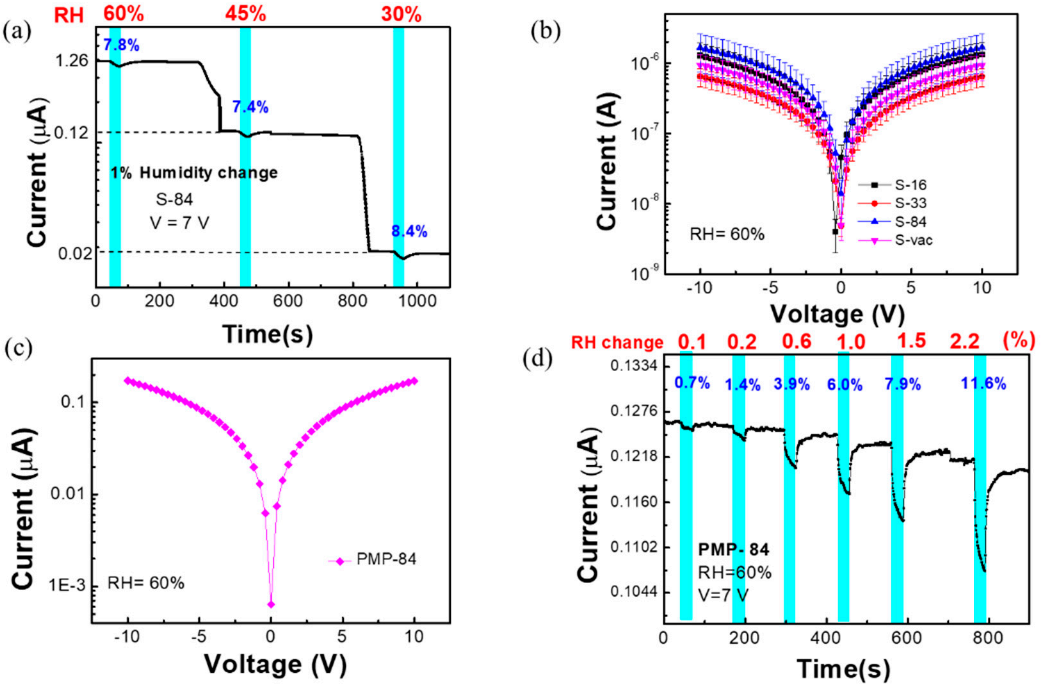

Figure S5. (a) Real time measurement of 1% RH changes under different relative humidity (b) I-V of different sensors (**S−16**, **S−33**, **S−84**, **S−vac**) under 60% RH (c, d) I-V and incremental-change of humidity response of **PMP−84** under 0.1% to 2.2% RH.

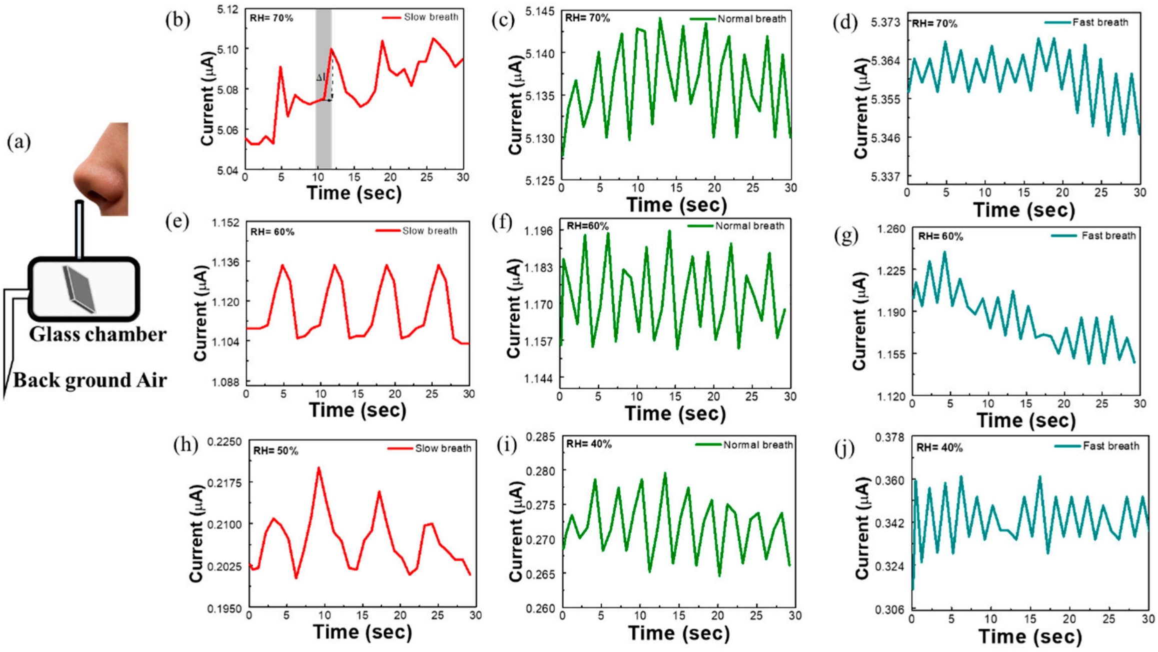

Figure S6. (a) Image of breath analysis inside glass chamber. The continuous slow, normal, and fast nasal breath was released from 5 cm away from substrates under different background relative humidity environment such as; (b-d) 70% RH (e-g) 60% RH, and (h-j) 50%.

Table S1. Recent flexible humidity sensor.

Material	Substrate (Thick.)	Deposition method	Anneal temperature	Dynamic range	Response (%)/ RH%	Res/Rec time	Reference
Graphene/ Ag colloids	PET (0.2-0.7 mm)	CVD	Infrared lamp	11-97	3.5/97	54/ 132 s	[17]
GNCP	PI (0.125 mm)	Drop cast	Evaporate close condition/ 12 h	0-90	20,00,000/90	20/17 ms	[22]
MWCNTs/ PLL	PI	Drop cast	60 °C/ 24 h	0-91.5	659.97/91.5 ~200/ 70	19/472 s	[23]
Cellulose	Cellulose paper	---	---	7.2-91.5	~3,50,000 /91.5	472/19 s,	[28]
SnS_2/rGO	PET (0.2-0.7 mm)	Printing	RT	11-97	96.92/ 97	6/15 s	[18]
Graphdiyne	PET	Ink Printing	---	0-50	140/43	0.29/ 0.36 s	[19]
Cellulose/ KOH	Cellulose	Hydrogel film	60 °C/ 24 h	11.3-97.7	NA	6/10.8 s	[27]
Cellulose NF/ Carbon black	PEN	Ink Printing	120 °C/ 5 m	30-90	120/90	10/6 s	[24]
MXene/ MWCNT	MWCNT Fabric	Drop cast	UV light	10-90	265/90	28/66 s (at 50% RH)	[30]
SnO_2	Plastic wrap (~10 µm)	Spin coating	26.2-40.8 °C / 1 m (NIR anneal)	15-70	3,24,000/ 70	90/ 150 s	**This work**
SnO_2	Plastic wrap	Spin coating	26.2-40.8 °C / 1 m (NIR anneal)	0.1-2.2	14/ 2.2	30/ 29 to 50 s	**This work**

Table S2. Recent metal oxide based humidity sensor under low curing temperature.

Material	substrate	Deposition method	Anneal temperature	Range	Response(%) / RH%	Res/Rec time	Reference
SnO_2	PET	Spin-spray	60 °C/ 1 h	5-95	72/95 60/70	69/47 s	[43]
In_2O_3/GO	Epoxy	Drop cast	60 °C / 4 h	11-97	8,44,200/97 ~15,000/70	15/ 2.5 s	[26]
In_2O_3	Epoxy	Drop cast	60 °C/ 4 h	11-97	2,11,025/97 ~13,500/70	38/ 3 s	[26]
Au/ZnO ZnO	Ceramic	Drop cast	60 °C/ 1 h	11-95	~100/95 ~90/95	16/28 s 36/44 s	[35]
Cu/Cu$_x$O	PC	Laser direct writing	<100 °C	0-55 55-95	60/55 100/95	3/23 s	[25]
SnO_2/rGo	PI	Sputter	60 °C/ 1 h	11-95	1,51,000/90	80/4 s	[41]
gC_3N_4/ ZnO	PI	Drop cast	60 °C / 12 h	11-95	10,50,000/95 ~99,900/70	22/5 s	[42]
ZnO	PI	Drop cast	60 °C / 12 h	11-95	381/95	22/5 s	[42]
ZnO/ SnO_2	---	Paste	60 °C / 12 h	11-95	12,25,361/95 ~10,000/70	35/8 s	[44]
SnO_2	---	Paste	60 °C / 12 h	11-95	40,906/95	--	[44]
SnO_2	Plastic wrap	Spin coating	NIR anneal 26-40 °C / 1 m	15-70	3,24,000/ 70	90/ 150 s	**This work**
SnO_2	Plastic wrap	Spin coating	NIR anneal 26-40 °C / 1 m	0.1-2.2	14/ 2.2	30/ 29 to 50 s	**This work**

Table S3. Fabrication of plastic wrap sensor under different annealing condition.

Plastic wrap (DI water: Ethanol)	Affordable temp. (ºC)	Sensor Name	NIR annealing	Vacuum annealing
**HDPE/resin (1:1)**	-46~80	S−16	16 W/cm^2 (1 m)	30 min
		S−33	33 W/cm^2 (1 m)	30 min
		S−84	84 W/cm^2 (1 m)	30 min
		S−vac	NA	60 min
**LDPE (4:1)**	-60~110	LDPE−84	84 W/cm^2 (1 m)	60 min
**PMP (1:3)**	-20~160	PMP−84	84 W/cm^2 (1 m)	60 min

Table S4. Atomic concentration (%) measured by XPS.

Different annealed film	C-1s	O-1s	Sn-3d	n=[O]/[Sn] in (SnO$_n$)
16 W/cm^2 **(S-16)** (HDPE/resin)	65.69	23.41	10.90	2.14
33 W/cm^2 **(S-33)** (HDPE/resin)	66.24	23.08	10.69	2.15
84 W/cm^2 **(S-84)** (HDPE/resin)	65.36	23.70	10.94	2.16
Vacuum **(S-vac)** (HDPE/resin)	70.82	20.17	9.02	2.23
200 °C (glass)	25.61	50.54	23.85	2.12
350 °C (glass)	16.13	55.61	28.27	1.97
500 °C (glass)	15.78	55.93	28.29	1.97
